# Advancing hospital-onset bacteraemia surveillance: a five-year retrospective study following the hospital-wide implementation of an automated surveillance system at a German university hospital

**DOI:** 10.1186/s13756-026-01708-9

**Published:** 2026-02-06

**Authors:** Ferenc Darius Rüther, Michael Behnke, Luis Alberto Peña Diaz, Frank Schwab, Christine Geffers, Seven Johannes Sam Aghdassi

**Affiliations:** 1 Institute of Hygiene and Environmental Medicine, Charité—Universitätsmedizin Berlin, Corporate Member of Freie Universität Berlin and Humboldt-Universität Zu Berlin, Hindenburgdamm 27, 12207 Berlin, Germany; 2 BIH Charité Digital Clinician Scientist Program, Berlin Institute of Health at Charité—Universitätsmedizin Berlin, BIH Biomedical Innovation Academy, Berlin, Germany

**Keywords:** Hospital-onset bacteraemia and fungaemia, Automated surveillance, Electronic health record, Retrospective study, Hospital epidemiology

## Abstract

**Background:**

Hospital-onset bacteraemia and fungaemia (HOB) has emerged as a novel surveillance metric in recent years and a prime target for automation of surveillance of healthcare-associated infections. However, real-life HOB data from European institutions remain scarce. This study explores the epidemiology of HOB at a German university hospital and describes characteristics of HOB cases.

**Methods:**

A retrospective single-centre study was conducted by applying an extended version of the Providing a Roadmap for Infection Surveillance in Europe (PRAISE) automated HOB algorithm to data from the electronic health records of all in-hospital patients admitted to Charité university hospital between 2018 and 2022. HOB rates per 1,000 patient days were calculated for different groups of wards. Furthermore, the distribution of microorganisms, share of antimicrobial resistance, and source of possible secondary HOB (defined as HOB-causing pathogens detected in relevant clinical samples other than blood) were analysed. Additionally, patient characteristics and outcomes were investigated.

**Results:**

A total of 3,648,254 patient days and 7,256 HOB with 8,357 microorganisms were included. The pooled HOB rate was 6.0 per 1,000 patient days in intensive care units, and between 0.9 and 2.0 in the various groups of non-intensive care units. Around 34.5% (n = 2,505) of HOB were deemed potentially secondary, with respiratory tract (37.6%, n = 943) being the most common source. A total of 1,106 of 8,357 (13.2%) microorganisms were classified as multidrug-resistant, including 60.5% (23 of 38) of *Acinetobacter baumannii* with resistance to carbapenems. Case fatality within 14 days of HOB onset was 16.2% (990 of 6,093 patients).

**Conclusions:**

Analysis of electronic health record data provides important insights into the epidemiology and characteristics of HOB cases. Substantial rates of antimicrobial resistance and case fatality underscore the relevance of HOB as an IPC metric. Results from this study may inform refinement of algorithms for automated HOB surveillance.

**Supplementary Information:**

The online version contains supplementary material available at 10.1186/s13756-026-01708-9.

## Background

Surveillance of healthcare-associated infections (HAI) is an integral component of infection prevention and control (IPC) and an effective means to reduce HAI rates [1–3]. Traditional manual surveillance requires manual review of a large number of patient files. As a result, it is prone to subjective interpretation and resource-intensive, usually limiting surveillance activities to selected hospital units [4]. Consequently, data from manual surveillance often lack the comprehensiveness needed to draw conclusions about the entire hospital population.

Automated surveillance is frequently seen as a means to close this gap, enabling the monitoring of the entire hospital population in real-time, increasing objectivity and reproducibility, and decreasing human workload [5]. Automated surveillance may also induce a shift to novel surveillance metrics, with hospital-onset bacteraemia and fungaemia (HOB) having been discussed as a primary use case for automation [6].

Recent studies have explored predictors, benchmarking methods, validity and clinical outcomes of HOB [7–13]. The HOB working group of the *Providing a Roadmap for Automated Infection Surveillance in Europe* (PRAISE) network recently published an article outlining the specifics of an automated HOB algorithm jointly developed by several European institutions. The publication also provided first insights into the HOB epidemiology of four European university hospitals [14]. Data from our institution was included in this analysis. Now, we want to perform a deeper analysis of our institution’s HOB data, by incorporating aspects such as antimicrobial resistance, sources of HOB, as well as characteristics and outcomes of patients with HOB into the analysis. Our goal is to provide a more detailed description of HOB epidemiology that will guide algorithm refinement, enhance its use in IPC practice, and inform future case–control studies.

## Methods

### Study design and setting

This retrospective single-centre study was conducted by applying the PRAISE HOB algorithm to the records of patients admitted to Charité university hospital between January 1, 2018 and December 31, 2022. A data export performed on February 23, 2024 was utilised.

We performed a secondary analysis of routinely collected clinical data. Data from the hospital electronic health records and the laboratory system were migrated into the HygienePortal, a digital IPC system of the hospital. It includes a data warehouse with structured data about patient movements (e.g., hospital admission and discharge, ward transfers), microbiological results (e.g., positive blood cultures), patient demographics (e.g., age, gender, comorbidities), and outcomes (e.g., in-hospital death). The individual variables are described below in detail.

The study included all in-hospital admissions of any age with a valid date of admission and discharge (or death), from all included areas of the hospital. In patients repeatedly admitted during the study period, each admission was counted as a unique patient case. Specific wards, e.g., psychiatry and emergency short-term care units, were excluded from the analysis, as these areas were deemed not suitable for HOB surveillance. Unlike in the previously mentioned publication on HOB epidemiology by the PRAISE network [14], we did not exclude paediatric patients. Potential additional differences between the current data and those reported previously, could be attributable to variations in the timing of data export.

### The HOB algorithm

The PRAISE HOB algorithm was utilised. The specifics of the algorithm have previously been described in detail [14]. Here, we provide a simplified overview of the algorithm’s workings. A positive blood culture with a pathogen taken on day 2 or later after hospital admission (admission = day 0) that was not already present on day 0 or day 1 constituted a HOB episode. For common commensals (as defined by the American National Healthcare Safety Network [15]), a confirmatory culture yielding the same microorganism within two days of the first positive blood culture was necessary. Repeat isolates showing the same microorganism within 14 days of HOB onset, did not constitute a new episode. Multiple HOB episodes occurring within two days of each other, were grouped into polymicrobial HOB. HOB episodes were attributed to the ward where the patient was two days prior to the start of the episode. HOB surveillance ended with discharge or death.

As an addition to the PRAISE algorithm, we attempted to algorithmically determine the potential source of infection. HOB where at least one microorganism was also detected in other relevant clinical materials in the period of 13 days before to three days after HOB onset, were considered potentially secondary HOB. This attribution was only done for HOB with pathogens, not for HOB caused by common commensals. According to the sampling material, possible secondary HOB were grouped into the following anatomical sites: respiratory tract, abdominal cavity, urinary tract, wound/surgical site, joints/bones/soft tissue, central nervous system and other/uncertain. HOB with more than one site were counted for all identified sites. All other HOB were presumed primary HOB.

### Included variables and considered outcomes

We calculated HOB rates per 1,000 patient days for the entire hospital, and separately for groups of wards. We categorised wards into five different groups: adult intensive care units (ICU), adult medical non-ICU (MED), adult surgical non-ICU (SUR), adult non-ICU other than medical or surgical (OTH), and paediatric wards including paediatric ICU and neonatal wards (PED). Besides the overall HOB rate, individual rates for HOB with pathogens, HOB with common commensals and polymicrobial HOB were calculated.

The causative microorganism for HOB were recorded and analysed. In case of polymicrobial HOB, all microorganisms were counted separately. HOB caused by *Acinetobacter baumannii*-group, Enterobacterales, *Enterococcus faecium*, *Enterococcus faecalis*, *Pseudomonas aeruginosa* and *Staphylococcus aureus* were evaluated for relevant antimicrobial resistance. The combination of antimicrobial marker and microorganism was similar to that of the protocol of the point prevalence survey of healthcare-associated infections and antimicrobial use in European acute care hospitals by the European Centre for Disease Prevention and Control [16]. The following pathogen-drug combinations were considered as HOB with multidrug-resistant organisms (MDRO):

- For *A. baumannii*-group, resistance to carbapenems (imipenem or meropenem)
- For Enterobacterales, resistance to third-generation cephalosporins (ceftazidime or cefotaxime) and/or carbapenems (imipenem or meropenem (for enterobacterales with intrinsic imipenem resistance, only meropenem was considered))
- For *E. faecium* and *E. faecalis*, resistance to vancomycin and/or linezolid
- For *P. aeruginosa*, resistance to carbapenems (imipenem or meropenem)
- For *S. aureus*, resistance to oxacillin (MRSA)

The algorithm processed antimicrobial susceptibility data based on categorical results (S/I/R) as reported by the laboratory information system. Polymicrobial HOB with both MDRO and susceptible organisms (e.g., MRSA and non-MDRO *Escherichia coli*) were counted as MDRO HOB.

For patients with HOB, comorbidities as well as invasive procedures and operations, were recorded by extracting data on *International Statistical Classification of Diseases and Related Health Problems (ICD)* version 10 and the German *Operation and Procedure Code (Operationen- und Prozedurenschlüssel, OPS)* version 2023 codes from the electronic health records [17]. ICD codes were used to calculate the Charlson comorbidity index (CCI) [18, 19] and to assess for the presence of sepsis during the course of admission. The details of the considered codes and the applied grouping of procedures and operations are illustrated in supplementary Table S1. In-hospital case fatality after HOB was recorded, but relatedness or causality was not assessed.

### Statistical analysis

Descriptive data of HOB and causative microorganisms are presented as aggregated results stratified by the different ward groups. Patient characteristics are depicted descriptively for the overall cohort. To investigate the outcome in-hospital death after HOB, patients with HOB were grouped into two cohorts (in-hospital death within 14 days of HOB onset vs. no in-hospital death within 14 days of HOB onset). In patients with multiple HOB episodes, only the first HOB was considered. Differences between the groups were tested using chi-squared test (for categorical variables) or Mann–Whitney-U test (for continuous variables). To investigate HOB-specific risk factors (e.g., species) for in-hospital death within 14 days of HOB onset, we used Cox-regression with a stepwise forward approach, adjusting for patient-related factors as potential confounders. A p-value < 0.05 was used as the threshold for statistical significance. Analyses were performed in SPSS version 29.0 (IBM Corp., Armonk, NY, USA).

### Ethical considerations

The study was approved by the Charité University Medicine Institutional Review Board (process number: EA2/060/23). Informed consent was not needed for this retrospective analysis of routinely collected data for surveillance purposes.

## Results

The total number of microorganisms isolated in blood cultures was 45,942. Through application of the algorithm, 7,256 HOB episodes were identified. Supplementary Figure S1 illustrates the data processing and annotation by the algorithm.

Altogether, 3,648,254 patient days were recorded, corresponding to an overall HOB rate of 2.0 per 1,000 patient days (Table 1).

**Table 1:** Rates of hospital-onset bacteraemia and fungaemia per ward group during the study period (2018–2022)

Parameter	Category	Total (n = 135)	ICU (n = 22)	MED (n = 51)	SUR (n = 35)	OTH (n = 11)	PED (n = 16)
No. of patient days	Total	3,648,254	448,148	1,396,811	1,113,151	331,238	358,906
No. of HOB episodes	Total	7256	2689	2775	966	474	352
HOB rate (per 1000 patient days)	Pooled mean	2.0	6.0	2.0	0.9	1.4	1.0
	Median (IQR)	1.5 (0.4–2.8)	5.1 (3.6–8.3)	1.4 (0.8–2.5)	0.7 (0.3–1.4)	1.0 (0.2–1.9)	0.7 (0.3–1.5)
No. of in-hospital days until onset of HOB	Median** (IQR)	14 (7–30)	19 (9–38)	12 (6–22)	11 (5–24)	15 (6–33)	13 (6–34)
No. of in-hospital days until onset of first HOB*	Median** (IQR)	12 (6–22)	14 (7–27)	11 (5–19)	9 (4–19)	12 (6–25)	10 (6–24)
No. of polymicrobial HOB episodes	Total (% of all HOB)	882 (12.2)	316 (11.8)	340 (12.2)	127 (13.1)	67 (14.1)	32 (9.1)
Polymicrobial HOB rate (per 1000 patient days)	Pooled mean	0.2	0.7	0.2	0.1	0.2	0.1
	median (IQR)	0.1 (0–0.4)	0.6 (0.3–1.1)	0.1 (0–0.3)	0.1 (0–0.2)	0.1 (0–0.3)	0.1 (0–0.1)
No. of HOB episodes with CC	Total (% of all HOB)	1396 (19.2)	529 (19.7)	561 (20.2)	171 (17.7)	103 (21.7)	32 (9.1)
CC HOB rate (per 1000 patient days)	Pooled mean	0.4	1.2	0.4	0.2	0.3	0.1
	Median (IQR)	0.2 (0.1–0.6)	1.1 (0.6–1.5)	0.2 (0.1–0.4)	0.1 (0–0.2)	0.1 (0–0.4)	0.1 (0–0.1)
No. of HOB episodes with pathogen	Total (% of all HOB)	6047 (83.3)	2231 (83.0)	2296 (82.7)	811 (84.0)	385 (81.2)	324 (92.0)
Pathogen HOB rate (per 1000 patient days)	Pooled mean	1.7	5.0	1.6	0.7	1.2	0.9
	Median (IQR)	1.3 (0.3–2.5)	4.5 (2.6–6.8)	1.1 (0.7–2.0)	0.6 (0.3–1.2)	0.9 (0.1–1.6)	0.6 (0.3–1.3)
No. of possible secondary HOB	Total (% of all HOB)	2505 (34.5)	1178 (43.8)	682 (24.6)	391 (40.5)	157 (33.1)	97 (27.6)

The data in this table pertains only to the wards included in the study. Multiple HOB with different microorganisms occurring within a two-day period were counted as a single polymicrobial HOB. Since polymicrobial HOB could involve both pathogens and common commensals, the total of HOB with pathogens and common commensals may exceed the overall number of HOB

* = In patients with multiple HOB during an admission, only the first HOB was considered

** = Here, the median does not pertain to the values of wards, but patients

CC, common commensal; HOB, hospital-onset bacteraemia and fungaemia; ICU, adult intensive care unit; IQR, interquartile range; MED, adult medical non-intensive care ward; OTH, adult non-intensive care ward other than medical or surgical; PED, paediatric ward; SUR, adult surgical non-intensive care ward

The HOB rate in ICU was 6.0 (pooled mean) compared to 0.9–2.0 in non-ICU. The proportions of polymicrobial HOB (11.7–14.1%), HOB with common commensal (17.7–21.7%) and HOB with pathogen (81.2–84.0%) were similar between all adult ward groups, but different for paediatric wards. Here, the algorithm detected lower proportions of polymicrobial HOB (9.1%) and HOB with common commensal (9.1%), and a higher proportion of HOB with pathogen (92.0%). The share of possible secondary HOB among all HOB was 34.5% (2,505 of 7,256 HOB), and was considerably lower for MED (24.6%) than for SUR (40.5%) and ICU (43.8%). When differentiating possible secondary HOB by anatomical source, respiratory tract (37.6%, n = 943 of 2,505) and urinary tract (33.7%, n = 845) were found to be most common (supplementary Figure S2).

**Fig. 1:**
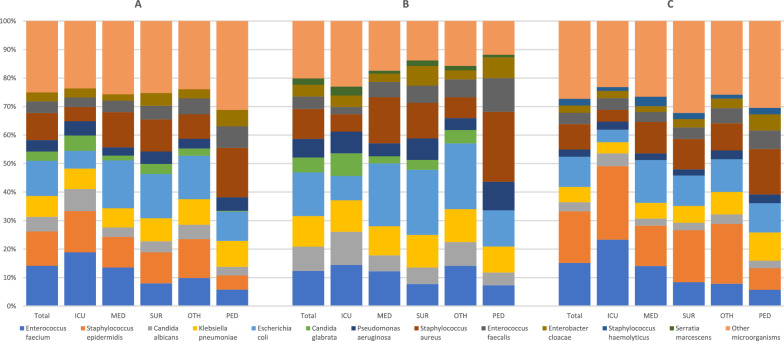
The ten most frequent microorganisms in hospital-onset bacteraemia and fungaemia (HOB) total and per ward group. Footnote: all HOB (**A**), possible secondary HOB (**B**) and presumed primary HOB (**C**). The total number of 8,357 microorganisms is distributed among adult intensive care units (ICU, n = 3,067), adult medical non-intensive care wards (MED, n = 3,207), adult surgical non-intensive care wards (SUR, n = 1,128), adult non-intensive care wards other than medical or surgical (OTH, n = 557), and paediatric wards (PED, n = 398). The classification as the ten most frequent microorganisms pertains to the overall hospital. Within the displayed individual groups of wards, non-displayed microorganisms might be among the ten most frequent microorganisms for the respective group. For a consistent presentation of results, these were not included in the figure

Overall, 8,357 microorganisms were recorded in 7,256 HOB episodes. The most frequent microorganisms were *E. faecium* (14.1%), *E. coli* (12.3%) and *Staphylococcus epidermidis* (12.0%). When distinguishing by ward group, considerable variation was observed. For instance, the share of *E. faecium* was highest in ICU (18.9%), where it was the most frequent causative microorganism, and lowest in PED (5.8%). Conversely, *S. aureus* was found to be the most frequent causative microorganism in PED (17.3%). Further details regarding microorganism distribution by ward type are illustrated in Fig. 1.

Of 7,256 HOB, 1,089 (15.0%) were classified as MDRO HOB. At the microorganism level, 1,106 of 8,357 (13.2%) microorganisms in HOB episodes were classified as MDRO. The highest share of MDRO was detected for *A. baumannii*-group, with 23 of 38 isolates (60.5%) exhibiting resistance to carbapenems. Carbapenem resistance and third-generation cephalosporin resistance in Enterobacterales was found in 50 of 2,490 isolates (2.0%), and 459 of 2,490 isolates (18.4%) respectively. Carbapenem-resistant isolates accounted for 49 of 334 (14.7%) of all *P. aeruginosa* isolates. For *E. faecium* and *E. faecalis*, 479 of 1,526 (31.4%) of isolates had resistance to vancomycin, and 12 of 1,526 (0.8%) to linezolid. Finally, 80 of 795 (10.1%) *S. aureus* isolates were classified as MRSA. When comparing MDRO patterns across ward groups, considerable differences were noted with ICU exhibiting the highest proportion of resistance for all considered microorganisms (Table 2).

**Table 2:** Multidrug-resistant organisms in hospital-onset bacteraemia and fungaemia episodes per ward group

Microorganism	Resistance marker	Total			ICU			MED			SUR			OTH			PED		
		All	MDRO (n)	MDRO (%)	All	MDRO (n)	MDRO (%)	All	MDRO (n)	MDRO (%)	All	MDRO (n)	MDRO (%)	All	MDRO (n)	MDRO (%)	All	MDRO (n)	MDRO (%)
*Acinetobacter baumannii*	Carbapenems	38	23	60.5	24	21	87.5	6	0	0	3	1	33.3	4	1	25.0	1	0	0
Enterobacterales	Third-generation cephalosporins	2490	459	18.4	740	160	21.6	990	175	17.7	422	71	16.8	188	28	14.9	150	25	16.7
	Carbapenems		50	2.0		22	3.0		20	2.0		5	1.2		1	0.5		2	1.3
*Enterococcus faecium, Enterococcus faecalis´*	Vancomycin	1526	479	31.4	681	281	41.3	562	145	25.8	144	28	19.4	86	17	19.8	53	8	15.1
	Linezolid		12	7.9		8	1.2		4	0.7		0	0		0	0		0	0
*Pseudomonas aeruginosa*	Carbapenems	334	49	14.7	154	29	18.8	93	14	15.1	49	4	8.2	19	1	5.3	19	1	5.3
*Staphylococcus aureus*	Oxacillin	795	80	10.1	153	20	13.1	397	35	8.8	128	15	12.5	48	5	10.4	69	5	7.2

ICU, adult intensive care unit; IQR, interquartile range; MED, adult medical non-intensive care ward; MDRO, multidrug-resistant organism; OTH, adult non-intensive care ward other than medical or surgical; PED, paediatric wards; SUR, adult surgical non-intensive care ward

In total, 6,093 patients developed at least one HOB between 2018 and 2022, with 819 (13.4%) patients having two or more episodes. The majority of affected patients were male (62.3%), the median age was 64 years (interquartile range, IQR: 53–74), and the median CCI was 5 points (IQR: 3–8). The frequency of underlying conditions considered for the CCI in patients with HOB is depicted in supplementary Figure S3. Of all patients with HOB, 39.3% were admitted as an emergency, and in 7.0% a community-onset bacteraemia or fungaemia had been detected prior to the HOB. An OPS-code for an invasive procedure between admission and onset of first HOB was recorded in 71.4% of patients, and a code indicating surgery in 47.1% of patients. Overall, 1,728 (28.4%) of patients died in-hospital, and 57.3% (n = 990) of deaths occurred within 14 days of HOB onset, or first HOB in patients with multiple HOB episodes, resulting in a case fatality rate of 16.2% within 14 days. Further details about patient characteristics and outcomes are depicted in Table 3.

**Table 3:** Characteristics of patients with hospital-onset bacteraemia and fungaemia (N = 6093)

Parameter	Patients with HOB (n = 6093)	
	Number/Median	%/IQR
*Gender*		
Female	2295	37.7
Male	3798	62.3
Age	64	53–74
Charlson Comorbidity Index	5	3–8
*Type of hospital admission*		
Via emergency department	2392	39.3
Elective	3701	60.7
Length of stay before HOB in days	11	5–21
*Ward specialty attributed to HOB*		
ICU	2027	33.3
MED	2474	40.6
SUR	875	14.4
OTH	412	6.7
PED	305	5.0
Community-onset bacteraemia	425	7.0
Invasive procedure between admission and onset of HOB	4348	71.4
*Type of invasive procedure between admission and onset of HOB*		
Central venous catheter insertion	2821	46.3
Punctures & catheterisation (e.g. cardiac)	1443	23.7
Ventilation	1427	23.4
Dialysis	1222	20.1
Drainage, aspiration, lavage	1147	18.8
Biopsy with or without incision	1083	17.8
Extracorporeal membrane oxygenation	358	5.9
Endoscopy	332	5.4
Surgery between admission and onset of HOB	2870	47.1
*Type of surgery between admission and onset of HOB*		
Abdominal surgery	1334	21.9
Cardiovascular surgery	849	13.9
Respiratory surgery	812	13.3
Skin and soft tissue surgery	547	9.0
Orthopaedic and trauma surgery	391	6.4
Urological surgery	310	5.1
Neurological surgery	339	5.6
Gynaecological and obstetric surgery	87	1.4
Other surgeries	310	5.1
*Outcome after HOB*		
Additional HOB during hospital stay	819	13.4
ICU admission associated with HOB*	1388	22.8
ICD code “sepsis” in patient record	3312	54.5
In-hospital death within 14 days of onset of HOB	990	16.2
In-hospital death within hospital stay	1728	28.4

In patients with multiple HOB episodes, only the first HOB was considered

*ICU admissions three days before until four days after onset of HOB were defined as admissions associated with HOB

HOB, hospital-onset bacteraemia and fungaemia; ICD, International Statistical Classification of Diseases and Related Health Problems; ICU, adult intensive care unit; IQR, interquartile range; MED, adult medical non-intensive care ward; OTH, adult non-intensive care ward other than medical or surgical; PED, paediatric ward; SUR, adult surgical non-intensive care ward

A univariable analysis of characteristics of HOB patients with (n = 990) and without (n = 5,103) in-hospital death within 14 days of HOB onset, demonstrated differences between the two groups. For instance, patients with in-hospital death within 14 days tended to be older (share of patients over 65 years: 57.4% vs. 44.4%, p < 0.001) and were admitted via the emergency department significantly more often (44.2% vs. 38.3%, p < 0.001) than those without in-hospital death within 14 days. The percentage of patients with chronic liver, heart and vascular diseases, and with invasive procedures before HOB was higher in the group with in-hospital death within 14 days. Concerning surgical procedures, no significant differences were found. When differentiating case fatality rates of patients with HOB by the causative organism, the group with in-hospital death within 14 days had a significantly higher share of HOB with pathogens, while the share of HOB with common commensals was significantly lower. Among the most frequent microorganisms, HOB with *Candida albicans* (29.2%), *P. aeruginosa* (21.3%), *E. faecium* (20.8%), and *K. pneumoniae* (17.3%) were found to have rates of associated in-hospital case fatality above the 14 day overall average (16.2%). Likewise, HOB with MDRO had an associated case fatality rate of 18.2%. Supplementary Table S2 summarises the results of the univariable analysis.

**Fig. 2:**
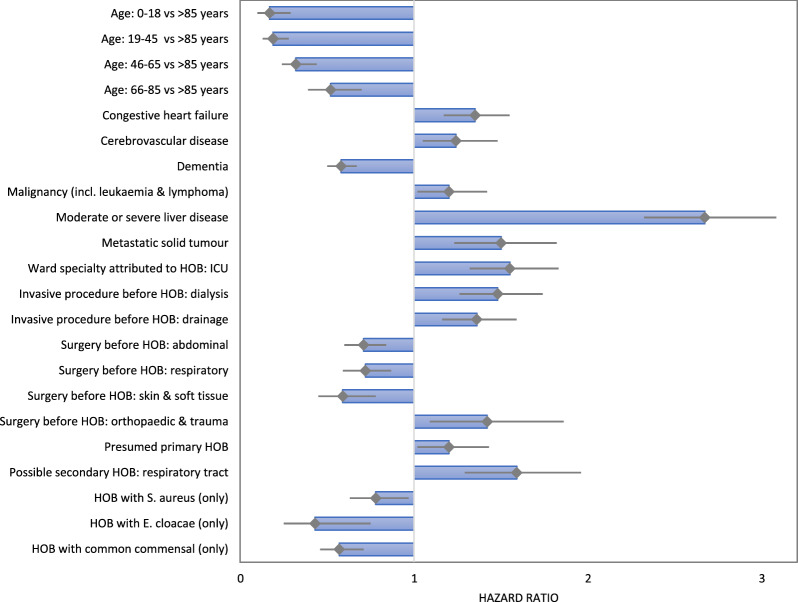
Results of the multivariable Cox-regression analysis for factors associated with in-hospital death within 14 days of onset of hospital-onset bacteraemia and fungaemia. Footnote: Only statistically significant results are displayed. In patients with multiple HOB episodes, only the first HOB was considered. HOB episodes were attributed to the ward where the patient was two days prior to the start of the episode. Possible secondary HOB entail the detection of the causative microorganism of HOB in other relevant clinical materials in the period 13 days before to 3 days after onset of HOB episode. Grey diamond sign = hazard ratio, grey lines = 95% confidence intervals of hazard ratios, blue boxes = magnitude of the hazard ratios (for illustrative purposes only, no additional statistical meaning). Abbreviations: HOB: hospital-onset bacteraemia and fungaemia; ICU: adult intensive care unit

The Cox-regression analysis identified several factors that were significantly associated with decreased or increased risk of in-hospital death within 14 days of HOB onset (Fig. 2).

The highest hazard ratios were observed for HOB that occurred in patients who had moderate or severe liver disease (2.67) or had undergone dialysis treatment (1.48) or orthopaedic / trauma surgery (1.42) before HOB. HOB caused by common commensals as opposed to HOB caused by pathogens were associated with a lower hazard ratio (0.57), although low hazard ratios were also found for *E. cloacae* (0.43) and *S. aureus* (0.78). An association with increased mortality was also observed for HOB that were not identified as possible secondary (i.e. presumed primary HOB) (hazard ratio of 1.2). Among possible secondary HOB, HOB with the suspected source respiratory tract significantly increased the risk of in-hospital death within 14 days (hazard ratio of 1.59) (Supplementary Table S3).

## Discussion

By applying the PRAISE HOB algorithm and extracting additional patient-level data, this study enhances the understanding of HOB beyond the mere rate and reveals key epidemiological patterns and patient-specific factors.

The overall HOB rate observed in this study was comparable to previously published rates applying a different algorithm [8, 10, 13] and very similar to results from a Dutch study applying the PRAISE HOB algorithm in four different hospitals [20]. Unlike other investigations that limited their scope to the first HOB episode during a hospital stay [21], this study captured all episodes, identifying multiple HOB occurrences in 13% of cases. This finding highlights that certain patients experience multiple significant infection events during a hospital admission, underscoring the importance of longitudinal and continuous surveillance. By ceasing surveillance after the first infection, critical information about subsequent events may be lost. Of note, in 3.2% of cases, the same microorganism was detected as in a previous HOB episode, suggesting persistence rather than reinfection. Nonetheless, as ongoing bacteraemia can signal inadequate source control or treatment failure, such events are still relevant to IPC monitoring. Similarly, possible secondary HOB accounted for a substantial share of all HOB episodes in our study, while conventional surveillance approaches are often heavily focused on primary bloodstream infections, such as central-line-associated bloodstream infections [22]. When looking at the frequency of possible secondary HOB in our dataset, this conventional approach risks overlooking a significant portion of bloodstream infections. Possible secondary HOB was nearly twice as frequent in ICU patients compared to non-ICU settings. This finding could reflect both the complexity of ICU infections and the broader range of samples collected in ICU settings.

The most commonly identified sources of secondary HOB as per the definition applied, were respiratory tract, urinary tract, and wounds or surgical sites. The frequency of these sources mostly aligns with observations of other studies considering this aspect [11, 23–25]. For instance, in a study by Leekha et al. investigating over 1,700 HOB cases in 13 American hospitals, HOB with a gastrointestinal or abdominal source were found to be most common, followed by HOB with a respiratory and urinary source (when excluding endovascular and unknown sources that likely would not conform to our definition of secondary) [11]. Studies focusing on ICU by Ben-David et al. and Culshaw et al. in Israel and the United Kingdom respectively, identified the respiratory tract as the most common source of bloodstream infection [23, 25]. Importantly, differences in findings between our study and previous publications could also be attributed to methodological variations, as our study relied on algorithm-based source determination by using microbiological specimens from different body compartments as a proxy. Compared to strategies based on a comprehensive chart review, this increases reproducibility and potentially reduces subjectivity, but is likely less accurate. Accordingly, our method for secondary HOB must be viewed as somewhat experimental and requiring further validation. Additionally, variations in clinical settings and sampling routines further reduce comparability across institutions, highlighting the importance of accounting for local diagnostic practices in HOB surveillance.

HOB occurred across all ward types, including general wards and paediatric units, and was not restricted to ICU. While by absolute numbers HOB cases were more frequent in general wards, traditional surveillance methods often deprioritise these areas [6]. This finding emphasises the importance of incorporating HOB into broader infection surveillance frameworks to reduce blind spots. In paediatric wards, a lower proportion of polymicrobial and commensal organism-related HOB was observed, possibly due to more restrictive blood culture sampling protocols [26]. These results suggest that certain patient groups may require different approaches, indicating that the HOB algorithm might need adaptation to better address their specific characteristics. This is also reflected by the observed pronounced differences in the distribution of causative microorganisms between ward groups. Microorganism distributions can be a good access point when trying to derive action points from HOB data. Here, the high share of HOB caused by *S. aureus* in paediatric wards (17.3%) is a particularly interesting finding, given the outcomes and the preventive potential of *S. aureus* bacteraemia [11, 27].

A substantial proportion of HOB cases in this study were caused by MDRO, particularly carbapenem-resistant *A. baumannii*, which were predominantly identified in ICU. The carbapenem resistance rate of 88% for *A. baumannii* in ICU observed in this study, is consistent with findings from a study in Israel [28], but significantly exceeds the 20% rate reported for German ICU by the Robert Koch Institute between 2018 and 2022 [29]. These disparities highlight the importance of implementing the algorithm in a wider range of hospitals, including primary and secondary care centres, to generate more representative data. Additionally, the high prevalence of vancomycin-resistant enterococci and third-generation cephalosporin-resistant organisms was consistent with other German surveillance data [30]. Nonetheless, these findings must be interpreted cautiously given the likely overrepresentation of a severely ill patient population in this study, especially in ICU, who inherently have higher risks of acquiring nosocomial infections. Future linkage with antibiotic consumption data could also help to explain the prevalence of antimicrobial resistance.

Several comorbidities (e.g., chronic liver disease) and preceding invasive procedures (e.g., dialysis, orthopaedic/trauma surgery) were associated with increased mortality within 14 days of HOB onset, potentially reflecting underlying patient vulnerability rather than direct effects of HOB. The association of commensals with decreased risk was expected to some extent, given that commensal organisms, even when meeting the criteria for HOB based on confirmatory blood cultures, may occasionally reflect contamination rather than true infection [31]. This underscores the need for careful interpretation of cases involving common commensals to avoid misclassification. In contrast, the negative association between *S. aureus* HOB and case fatality was unexpected, given the grave clinical impact of *S. aureus* bloodstream infections [27]. However, similar findings have been reported in ICU populations [32]. This could also indicate a more alert response to suspicion or detection of *S. aureus* from blood cultures with consecutive faster initial treatment and general improvements in the management of *S. aureus* bloodstream infections. Notably, *S. aureus* HOB occurred comparatively early after admission (median 8 days vs. 14 days for all HOB), which may suggest that these infections developed at a stage when patients were less affected by complications of prolonged hospitalisation and overall morbidity. Possible secondary HOB linked to the respiratory tract were associated with increased risk of in-hospital death, possibly reflecting the negative impact of underlying lower respiratory tract infections. Somewhat surprisingly, HOB with MDRO were not significantly associated with a higher risk of in-hospital mortality in the Cox regression analysis. These findings provide initial insights, yet also highlight the need for future studies to explore acute clinical indicators more comprehensively.

Several limitations warrant consideration. This study was conducted at a single tertiary care university hospital, potentially biasing the findings toward a sicker-than-average patient population and limiting generalizability. Paediatric cases, spanning neonatal, paediatric ICU, and non-ICU wards, were combined into a single group to simplify analysis, despite the heterogeneity of this population. Moreover, the study relied exclusively on automated data extraction, with no manual validation of HOB cases. As such, the accuracy of patient characteristics, secondary HOB classification and ward attribution remains unverified. Future studies incorporating clinical chart reviews will be critical for refining the algorithm and confirming the findings reported here. In patients with multiple HOB episodes, the Cox-regression analysis only considered the first HOB episode to avoid duplicate counting, leaving the cumulative impact of multiple episodes on patient outcomes unassessed. Furthermore, repeated admissions were counted as separate patients, which may have introduced minor confounding. The outcome in-hospital death was investigated for the time period of 14 days after HOB onset, which may be too short in some instances. The true case fatality rate is likely higher than what we found in this analysis. Also, the analysis did not include a control group of patients without HOB. Therefore, a comparison of descriptive characteristics of patients with HOB with the overall inpatient population was not possible. Finally, analyses of risk factors for HOB-associated in-hospital death only considered a limited number of variables and were restricted by data availability. Results from this analysis report mere associations and do not allow for causal inferences or conclusions about the extent of additional mortality attributable to HOB. The study also did not evaluate the preventability of HOB or the adequacy of subsequent treatment. These aspects need to be addressed in future validation studies.

## Conclusion

This study highlights the potential of automated surveillance to enhance understanding of HOB epidemiology across clinical settings. Unlike conventional surveillance methods focused on ICU and central-line-associated bloodstream infections, automated systems capture both primary and secondary HOB, addressing critical blind spots. The high proportion of MDRO-associated HOB underscores its relevance for antimicrobial stewardship. Refining algorithms for specific populations, such as paediatric patients, and investigating risk factors for both HOB occurrence and adverse outcomes will further inform the usability of HOB as an automated surveillance metric.

## Supplementary Information

Supplementary Material 1

## Data Availability

Not applicable, because all data were collected within the context of the surveillance of healthcare-associated infections conducted in accordance with the German Protection against Infection Act.

## Abbreviations

CC: Common commensal
CCI: Charlson comorbidity index
CI: Confidence interval
CVC: Central venous catheter
ECMO: Extracorporeal membrane oxygenation
HAI: Healthcare-associated infection
HOB: Hospital-onset bacteraemia and fungaemia
ICD: International statistical classification of diseases and related health problems
ICU: Adult intensive care unit
IPC: Infection prevention and control
IQR: Interquartile range
MDRO: Multidrug-resistant organism
MED: Adult medical non-intensive care unit
MRSA: Methicillin-resistant *Staphylococcus aureus*
OPS: Operation and procedure code (German: Operationen- und Prozedurenschlüssel)
OTH: Adult non-intensive care unit other than medical or surgical
PED: Paediatric wards including intensive care units and neonatal wards
PRAISE: Providing a roadmap for automated surveillance in Europe
SUR: Adult surgical non-intensive care unit
