# Supplementary material for: Advancing hospital-onset bacteraemia surveillance: a five-year retrospective study following the hospital-wide implementation of an automated surveillance system at a German university hospital
Source: Antimicrob Resist Infect Control. 2026 Feb 6;15:28. doi: 10.1186/s13756-026-01708-9 (PMC12930965; doi:10.1186/s13756-026-01708-9)
Supplement: Supplementary file 1 — Supplementary Material 1 [file 13756_2026_1708_MOESM1_ESM.pdf]

Additional file 1

Online supplementary material to

Advancing hospital-onset bacteraemia surveillance: a five-year retrospective study following the hospital-wide implementation of an automated surveillance system at a German university hospital

Authors: Ferenc Darius Rüther[1], Michael Behnke[1], Luis Alberto Peña Diaz[1], Frank Schwab[1], Christine Geffers[1], Seven Johannes Sam Aghdassi[1,2]

[1] Charité – Universitätsmedizin Berlin, corporate member of Freie Universität Berlin and Humboldt-Universität zu Berlin, Institute of Hygiene and Environmental Medicine, Berlin, Germany

[2] Berlin Institute of Health at Charité – Universitätsmedizin Berlin, BIH Biomedical Innovation Academy, BIH Charité Digital Clinician Scientist Program, Berlin, Germany

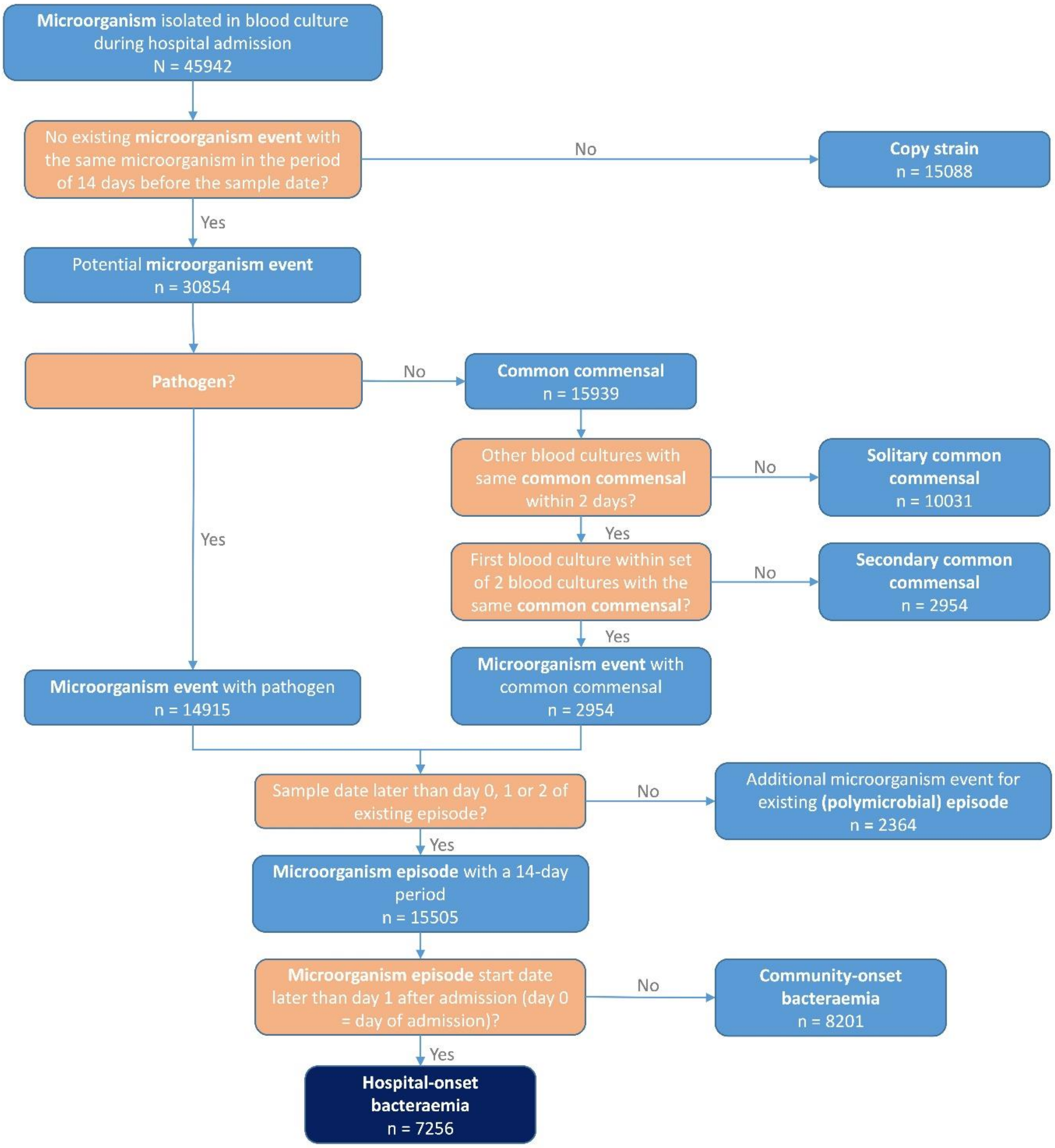

Supplementary Figure S1 Flowchart of the processing of positive blood cultures during the study period (2018-2022) by the PRAISE HOB algorithm

Footnote: The flowchart illustrates the relevant steps for the identification of hospital-onset bacteraemia and fungaemia (HOB) episodes. The specifics and terminology of the PRAISE HOB algorithm have been published in detail (doi: 10.1101/2024.09.16.24310433). A total of 48 microorganism episodes were excluded because they were attributed to excluded wards. Since the exclusion of wards is not a proprietary element of the algorithm, this is not illustrated in the flowchart.

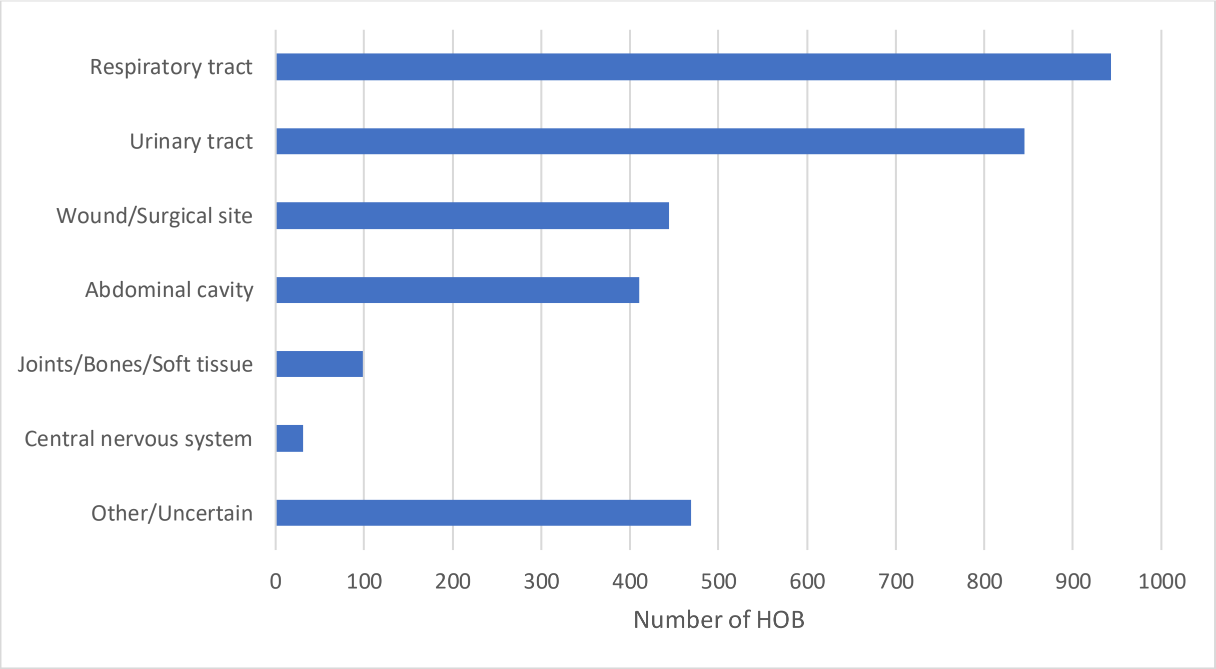

Supplementary Figure S2 Sources of possible secondary hospital-onset bacteraemia and fungaemia (HOB)

Footnote: Data from 2,505 HOB with microorganisms found in relevant clinical materials other than blood in the period 13 days before to 3 days after onset of HOB episode. HOB with more than one site were counted for all identified sites.

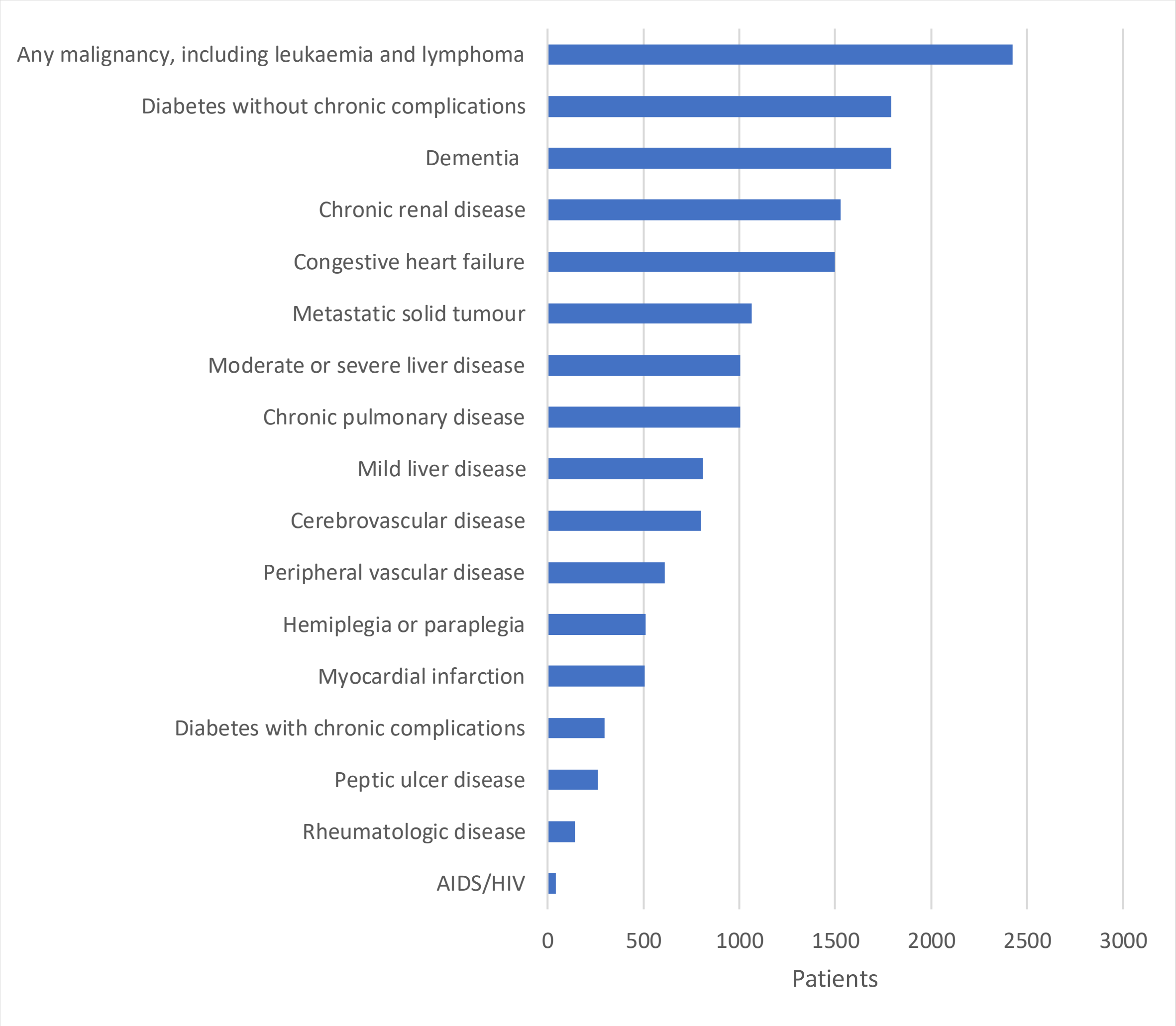

Supplementary Figure S3 Frequency of chronic diseases considered for the Charlson comorbidity index in 6,093 patients with hospital-onset bacteraemia and fungaemia (HOB)

Footnote: In 453 patients with HOB none of the chronic diseases were recorded. The details of the considered codes and the applied grouping of procedures and operations are illustrated in Supplementary Table S1.

Supplementary Table S1 ICD and OPS codes considered for the data analysis presented in the main manuscript

**All ICD codes grouped by the chronic diseases considered for the calculation of the Charlson comorbidity index, as indicated by Quan et al (DOI: 10.1097/01.mlr.0000182534.19832.83)**	
Myocardial Infarction	I21; I22; I25.2
Congestive Heart Failure	I09.9; I11.0; I13.0; I13.2; I25.5; I42.0; I42.5; I42.6; I42.7; I42.8; I42.9; I43; I50
Peripheral Vascular Disease	I70; I71; I72.1; I72.4; I73.1; I73.8; I73.9; I74.2; I74.4; I77.1; I79.2; K55.8; K55.9; Z95.9
Cerebrovascular Disease	G45; G46; H34.0; I6
Dementia	F00; F01; F02; F03; F05; G30; G31.0; G31.1; G31.2
Chronic Pulmonary Disease	E84; I27.0; I27.2; I27.8; I27.9; I84.1; I96.1; J40; J41; J42; J43; J44; J45; J46; J47; J60; J61; J62; J63; J64; J65; J66; J67; J68.4; J70.1; J70.3
Connective Tissue Disease Rheumatic	M05; M06; M31.3; M31.5; M32; M33; M34; M35.1; M35.5; M36.0
Peptic Ulcer Disease	K25; K26; K27; K28
Mild Liver Disease	B18; K70.0; K70.1; K70.2; K70.3; K70.9; K71.3; K71.4; K71.5; K71.7; K73; K74; K76.0; K76.1; K76.2; K76.3; K76.4; K76.8; K76.9; Z94.4
Diabetes without complications	E10.0; E10.1; E10.9; E11.0; E11.1; E11.9; E12.0; E12.1; E12.9; E13.0; E13.1; E13.9; E14.0; E14.1; E14.9
Diabetes with complications	E10.2; E10.3; E10.4; E10.5; E10.7; E10.8; E11.2; E11.3; E11.4; E11.5; E11.7; E11.8; E12.2; E12.3; E12.4; E12.5; E12.7; E12.8; E13.2; E13.4; E13.5; E13.7; E13.8; E14.2; E14.3; E14.4; E14.5; E14.7; E14.8
Paraplegia and Hemiplegia	G04.1; G11.4; G80.1; G80.2; G81; G82; G83.0; G83.1; G83.2; G83.4; G83.9
Renal Disease	I12.0; I13.1; N03.2; N03.3; N03.4; N03.5; N03.6; N03.7; N05.2; N05.3; N05.4; N05.5; N05.6; N05.7; N18; N19; N25.0; Z49.0; Z49.1; Z49.2; Z94.0; Z99.2
Cancer	C0; C1; C20; C21; C22; C23; C24; C25; C26; C30; C31; C32; C33; C34; C37; C38; C39; C40; C41; C43; C45; C46; C47; C48; C49; C50; C51; C52; C53; C54; C55; C56; C57; C58; C6; C70; C71; C72; C73; C74; C75; C76; C81; C82; C83; C84; C85; C88; C90; C91; C92; C93; C94; C95; C96; C97
Moderate or Severe Liver Disease	I85.0; I85.9; I86.4; I98.2; K70.4; K71.1; K72.1; K72.9; K76.5; K76.6; K76.7
Metastatic Carcinoma	C77; C78; C79; C80
AIDS/HIV	B20; B21; B22; B23; B24
**All ICD codes considered for the assignment of sepsis to patients**	
Sepsis	R65.1; R57.2; P36
**All OPS codes grouped by different types of procedures considered for the assignment of procedures to patients**	
Punctures & catheterization (e.g. cardiac)	1-204; 1-265; 1-27; 1-84; 1-85
Biopsy (with or without incision)	1-4; 1-5
Endoscopy	1-64; 1-69
Drainage, aspiration, lavage	8-14; 8-15; 8-16; 8-17
Ventilation	8-71
Central venous catheter insertion	8-831

Dialysis	8-851; 8-853; 8-854; 8-855; 8-856; 8-857; 8-85a
ECMO	8-852
**All OPS codes grouped by different types of surgery considered for the assignment of surgeries to patients**	
Neurological	5-01; 5-02; 5-03; 5-04; 5-05
Respiratory	5-32; 5-33; 5-34; 5-29; 5-30; 5-31
Cardiovascular	5-35; 5-36; 5-37; 5-38; 5-39
Abdominal	5-42; 5-43; 5-44; 5-45; 5-46; 5-47; 5-48; 5-49; 5-50; 5-51; 5-52; 5-53; 5-54
Urological	5-55; 5-56; 5-57; 5-58; 5-59; 5-60; 5-61; 5-62; 5-63; 5-64
Gynaecology & obstetric	5-65; 5-66; 5-67; 5-68; 5-69; 5-70; 5-71; 5-72; 5-73; 5-74; 5-75; 5-87; 5-88
Orthopaedic and trauma	5-78; 5-79; 5-80; 5-81; 5-82; 5-83; 5-84; 5-85; 5-86
Skin & soft tissue	5-89; 5-90; 5-91; 5-92
Other	5-06; 5-07; 5-08; 5-09; 5-10; 5-11; 5-12; 5-13; 5-14; 5-15; 5-16; 5-17; 5-18; 5-19; 5-20; 5-21; 5-22; 5-23; 5-24; 5-25; 5-26; 5-27; 5-28; 5-76; 5-77; 5-40; 5-41

The applied catalogue of codes was the 2023 version provided by The Federal Institute for Drugs and Medical Devices (Bundesinstitut für Arzneimittel und Medizinprodukte, BfArM). It can be downloaded from: https://www.bfarm.de/DE/Kodiersysteme/Services/Downloads/_node.html (Last access: 21/11/2024)

Supplementary Table S2 Univariable analysis of characteristics of patients with hospital-onset bacteraemia and fungaemia with in-hospital death and no in-hospital death within 14 days of onset

Parameter	In-hospital death within 14 days	No in-hospital death within 14 days	p-value	In-hospital case fatality rate (%)
Total Cases (n, %)	990 (100)	5103 (100)	NA	16.2
Gender (n, %)				
Female	367 (37.1)	1928 (37.8)	0.673	16.0
Male	623 (62.9)	3175 (62.2)	0.673	16.4
Age (n, %)				
0-18 years	20 (2.0)	289 (5.7)	<0.001	6.5
19-45 years	64 (6.5)	648 (12.7)	<0.001	9.0
46-65 years	338 (34.1)	1899 (37.2)	0.072	15.1
66-85 years	513 (51.8)	2110 (41.3)	<0.001	19.6
>85 years	55 (5.6)	157 (3.1)	<0.001	25.9
Charlson Comorbidity Index (median, IQR)	6 (4-8)	5 (3-7)	<0.001	NA
Comorbidities (n, %)				
Malignancy (incl. leukaemia and lymphoma)	397 (40.1)	2027 (39.7)	0.831	16.4
Diabetes without chronic complications	308 (31.1)	1481 (29.0)	0.195	17.2
Dementia	267 (27.0)	1522 (29.8)	0.073	14.9
Chronic renal disease	254 (25.7)	1275 (25.0)	0.660	16.6
Congestive heart failure	343 (34.6)	1157 (22.7)	<0.001	22.9
Metastatic solid tumour	193 (19.5)	870 (17.0)	0.067	18.2
Moderate or severe liver disease	331 (33.4)	676 (13.2)	<0.001	32.9
Chronic pulmonary disease	194 (19.6)	811 (15.9)	0.005	19.3
Mild liver disease	179 (18.1)	633 (12.4)	<0.001	22.0
Cerebrovascular disease	159 (16.1)	644 (12.6)	0.004	19.8
Peripheral vascular disease	132 (13.3)	481 (9.4)	<0.001	21.5
Hemiplegia or paraplegia	68 (6.9)	444 (8.7)	0.060	13.3
Myocardial infarction	114 (11.5)	394 (7.7)	<0.001	22.4
Diabetes with chronic complications	49 (4.9)	250 (4.9)	0.936	16.4
Peptic ulcer disease	39 (3.9)	221 (4.3)	0.667	15.0
Rheumatologic disease	24 (2.4)	119 (2.3)	0.819	16.8
AIDS/HIV	8 (0.8)	33 (0.6)	0.527	19.5
Admission via emergency department	438 (44.2)	1954 (38.3)	<0.001	31.5
Admission with community-onset bacteraemia (n, %)	86 (8.7)	339 (6.6)	0.021	20.2
Length of stay before HOB (n, %)				
<6 days	274 (27.7)	1500 (29.4)	0.276	15.4
6-11 days	258 (26.1)	1183 (23.2)	0.051	17.9
12-21 days	228 (23.0)	1198 (23.5)	0.762	16.0
>21 days	230 (23.2)	1222 (23.9)	0.629	15.8
Ward specialty attributed to HOB (n, %)*				
ICU	459 (46.4)	1568 (30.7)	<0.001	30.1
MED	345 (34.8)	2129 (41.7)	<0.001	13.9
SUR	117 (11.8)	758 (14.9)	0.013	13.4
OTH	47 (1.3)	365 (1.4)	0.006	11.4
PED	22 (0.8)	283 (4.1)	<0.001	7.2
Invasive procedure between admission and onset of HOB (n, %)	780 (78.8)	3568 (69.9)	<0.001	17.9
Type of invasive procedure between admission and onset of HOB (n, %)				
Central venous catheter insertion	553 (55.9)	2268 (44.4)	<0.001	19.6
Punctures & catheterization (e.g. cardiac)	289 (29.2)	1154 (22.6)	<0.001	20.0
Ventilation	278 (28.1)	1149 (22.5)	<0.001	19.5

Dialysis	322 (32.5)	900 (17.6)	<0.001	26.4
Drainage, aspiration, lavage	249 (25.2)	898 (17.6)	<0.001	21.7
Biopsy with or without incision	48 (4.8)	284 (5.6)	0.363	14.5
Extracorporeal membrane oxygenation	93 (9.4)	275 (5.4)	<0.001	25.3
Endoscopy	161 (16.3)	921 (18.0)	0.178	14.9
Surgery between admission and onset of HOB (n, %)	464 (46.9)	2406 (47.1)	0.889	16.2
Type of surgery between admission and onset of HOB (n, %)				
Abdominal surgery	193 (19.5)	1141 (22.4)	0.046	14.5
Cardiovascular surgery	153 (15.5)	696 (13.6)	0.131	18.0
Respiratory surgery	148 (14.9)	664 (13.0)	0.101	18.2
Skin and soft tissue surgery	73 (7.4)	474 (9.3)	0.054	13.3
Orthopaedic and trauma surgery	72 (7.3)	319 (6.3)	0.230	18.4
Urological surgery	32 (3.2)	278 (5.4)	0.004	10.3
Neurological surgery	56 (5.7)	283 (5.5)	0.889	16.5
Gynaecological and obstetric surgery	6 (0.6)	81 (1.6)	0.017	6.9
Other surgeries	29 (2.9)	280 (5.5)	<0.001	9.4
Polymicrobial HOB (n, %)	140 (14.1)	610 (12.0)	0.055	18.7
HOB with pathogen (n, %)	884 (89.3)	4119 (80.7)	<0.001	17.7
HOB with common commensal (n, %)	135 (13.6)	1121 (22.0)	<0.001	10.7
Possible secondary HOB (n,%)	391 (39.5)	1646 (32.3)	<0.001	19.2
Sources of possible secondary HOB (n, %)**				
Respiratory tract	203 (20.5)	518 (10.2)	<0.001	28.2
Urinary tract	107 (10.8)	620 (12.1)	0.233	14.7
Wound/Surgical site	49 (4.9)	315 (6.2)	0.143	13.5
Abdominal cavity	53 (5.4)	247 (4.8)	0.495	17.7
Central nervous system	3 (0.3)	27 (0.5)	0.352	10.0
Joints/Bones/Soft tissue	11 (1.1)	75 (1.5)	0.462	12.8
Other/Uncertain	68 (6.9)	314 (6.2)	0.390	17.8
Causative microorganism (n, %)				
Escherichia coli	150 (15.2)	788 (15.4)	0.817	16.0
Candida albicans	95 (9.6)	230 (4.5)	<0.001	29.2
Enterococcus faecium	180 (18.2)	686 (13.4)	<0.001	20.8
Klebsiella pneumoniae	86 (8.7)	410 (8.0)	0.492	17.3
Staphylococcus aureus	115 (11.6)	645 (12.6)	0.372	15.1
Staphylococcus epidermidis	103 (10.4)	796 (15.6)	<0.001	11.4
Pseudomonas aeruginosa	55 (5.6)	203 (4.0)	0.024	21.3
Enterococcus faecalis	34 (3.4)	260 (5.1)	0.026	11.6
Enterobacter cloacae	20 (2.0)	194 (3.8)	0.005	9.3
Other microorganisms	296 (29.9)	1523 (29.8)	0.973	16.3
HOB with MDRO (n, %)	148 (14.9)	664 (13.0)	0.101	18.2

All categorical parameters were analysed as dummy variables. P-values were calculated by chi-squared test (categorical variables) or Mann-Whitney-U test (continuous variables). In patients with multiple HOB episodes, only the first HOB was considered.

* = HOB episodes were attributed to the ward where the patient was two days prior to the start of the episode.

** = Causative microorganism of HOB also found in relevant clinical materials other than blood in the period 13 days before to 3 days after onset of HOB episode.

Abbreviations: HOB: hospital-onset bacteraemia and fungaemia; ICU: adult intensive care unit; IQR: interquartile range; MED: adult medical non-intensive care ward; MDRO: multidrug-resistant organism; OTH: adult non-intensive care ward other than medical or surgical; PED: paediatric wards; SUR: adult surgical non-intensive care ward

Supplementary Table S3 Results of the multivariable Cox-regression analysis for risk factors associated with in-hospital death within 14 days of onset of hospital-onset bacteraemia and fungaemia

**Parameter**	**Hazard ratio**	**95%-CI**	**p-value**
Age: 0-18 vs. >85 years	0.17	0.1-0.29	<0.001
Age: 19-45 vs. >85 years	0.19	0.13-0.28	<0.001
Age: 46-65 vs. >85 years	0.32	0.24-0.44	<0.001
Age: 66-85 vs. >85 years	0.52	0.39-0.7	<0.001
Congestive heart failure	1.35	1.17-1.55	<0.001
Cerebrovascular disease	1.24	1.05-1.48	0.014
Dementia	0.58	0.5-0.67	<0.001
Malignancy (incl. leukaemia & lymphoma)	1.2	1.02-1.42	0.029
Moderate or severe liver disease	2.67	2.32-3.08	<0.001
Metastatic solid tumour	1.5	1.23-1.82	<0.001
Ward specialty attributed to HOB: ICU*	1.55	1.32-1.83	<0.001
Invasive procedure before HOB: dialysis	1.48	1.26-1.74	<0.001
Invasive procedure before HOB: drainage	1.36	1.16-1.59	<0.001
Surgery before HOB: abdominal	0.71	0.6-0.84	<0.001
Surgery before HOB: respiratory	0.72	0.59-0.87	0.001
Surgery before HOB: skin & soft tissue	0.59	0.45-0.78	<0.001
Surgery before HOB: orthopaedic & trauma	1.42	1.09-1.86	0.001
Presumed primary HOB	1.2	1.02-1.43	0.033
Possible secondary HOB: respiratory tract**	1.59	1.29-1.96	<0.001
HOB with S. aureus (only)	0.78	0.63-0.97	0.026
HOB with E. cloacae (only)	0.43	0.25-0.75	0.003
HOB with common commensal (only)	0.57	0.46-0.71	<0.001

Only statistically significant results are displayed. Non-significant parameter considered in the multivariable analysis: admission via emergency department, ward specialty attributed to HOB: MED / SUR / OTH / PED, number of in-hospital days until onset of first HOB, comorbidities of the patient: diabetes / chronic renal disease / chronic pulmonary disease / mild liver disease / peripheral vascular disease / hemiplegia or paraplegia / myocardial infarction / peptic ulcer disease / rheumatologic disease / AIDS/HIV, invasive procedures before HOB: central venous catheter / punctures & catheterization / ventilation / biopsy / extracorporeal membrane oxygenation / endoscopy, surgery before HOB: cardiovascular / urological / neurological / gynaecological / other surgeries, sources of possible secondary HOB: urinary tract / wound/surgical site / abdominal cavity / central nervous system / joints/bones/soft tissue / other/uncertain source, polymicrobial HOB, HOB with E. faecium / E. coli / K. pneumoniae / C. albicans / E. faecalis / P. aeruginosa / C. glabrata / E. cloacae / other pathogen (only), HOB with MDRO

In patients with multiple HOB episodes, only the first HOB was considered. All parameters except age (categorical) and number of in-hospital days until onset of first HOB (continuous) were dummy coded.

* = HOB episodes were attributed to the ward where the patient was two days prior to the start of the episode.

** = Causative microorganism of HOB also found in relevant clinical materials other than blood in the period 13 days before to 3 days after onset of HOB episode.

Abbreviations: CI: confidence interval; HOB: hospital-onset bacteraemia ICU: intensive care unit
